# An Urgent Need for “Common Cold Units” to Study COVID-19

**DOI:** 10.4269/ajtmh.20-0246

**Published:** 2020-04-09

**Authors:** Scott B. Halstead

**Affiliations:** Consultant, North Bethesda, Maryland

Severe acute respiratory syndrome-2 (SARS-CoV-2) infections are causing a devastating mortality throughout the world in persons with preexisting health conditions. The pandemic has led to widespread quarantining, massive morbidity and mortality, suspension of social and business activities, and enormous economic loss. This terrible impact might be reduced if we can quickly find products to treat or prevent the disease.

A near-term possibility is treating novel coronavirus disease (COVID-19) with repurposed existing drugs^1^ or to use the antiviral remdesivir, a nucleotide analog under clinical investigation in China and elsewhere. Remdesivir has been shown to have efficacy against Middle East respiratory syndrome in a monkey model.^2^ New compounds will undoubtedly emerge from the laboratory. Antibodies offer promising options. Convalescent severe acute respiratory syndrome-1 antibodies administered early in acute illness were shown to reduce disease severity.^3^ Efforts are well underway to manufacture gamma globulin from COVID-19 convalescent sera or, alternatively, to derive monoclonal antibodies.^4,5^

Another realistic possibility is to use antibodies to protect the vulnerable population. After World War II, commercial gamma globulin was used to provide short-term protection against measles, paralytic poliomyelitis, hepatitis A, and hepatitis B.^6–10^ In the 1950s, a large-scale blinded efficacy trial found that gamma globulin given to 100,000 children successfully blunted attack rates of poliomyelitis paralysis.^8^ For COVID-19, antibodies or monoclonal antibodies can be given to prevent infection in high-risk persons, care givers, and healthcare workers. To avoid possible antibody-dependent enhancement of COVID-19 infections, the Fc terminus of IgG antibodies should be removed or inactivated.^11^

Tests for safety and efficacy of candidate vaccines and drugs begin in animal models and are completed in humans. For vaccines, the long process starts by demonstrating relevant immune responses in humans in the absence of unwanted side effects and culminates with evidence of protection in randomized, blinded trials in diseased populations. For antibody preparations designed to prevent COVID-19, the relationship between in vitro neutralization and prevention of SARS-CoV-2 infection in humans must be established. There is a long history of using human challenge models to establish candidate therapeutic and preventive products for microbial pathogens.^12–14^ Support has been voiced for using direct human challenge studies to shorten the time for COVID-19 vaccine approval.^15^ Fortunately, SARS-CoV-2 infections in young adults uncommonly produce severe disease. The virus has been adapted to grow in Vero cells.^16^ An interesting outcome of establishing a human model could be the discovery that tissue culture passage of SARS-CoV-2 reduces its pathogenicity.

There is precedent for direct studies on coronavirus infections in humans. In 1946, the British Medical Research Council recruited adult human volunteers to study the etiology, epidemiology, prevention, and treatment of common colds. Over a period of 40 years, thousands of adult volunteers, ages 18–54 years, recruited with advertisements to spend a 10-day “holiday” at the Common Cold Unit at Harvard Hospital, near Salisbury, were infected with preparations of cold viruses. To bring protective products on line in a matter of months to meet the current emergency, affected nations should establish national coronavirus clinical units.
